# Serological Evidence of Widespread Zika Transmission across the Philippines

**DOI:** 10.3390/v13081441

**Published:** 2021-07-23

**Authors:** Joseph R. Biggs, Ava Kristy Sy, Oliver J. Brady, Adam J. Kucharski, Sebastian Funk, Yun-Hung Tu, Mary Anne Joy Reyes, Mary Ann Quinones, William Jones-Warner, James Ashall, Ferchito L. Avelino, Nemia L. Sucaldito, Amado O. Tandoc, Eva Cutiongco-de la Paz, Maria Rosario Z. Capeding, Carmencita D. Padilla, Martin L. Hibberd, Julius Clemence R. Hafalla

**Affiliations:** 1 Department of Infection Biology, Faculty of Infectious and Tropical Diseases, London School of Hygiene and Tropical Medicine, London WC1E 7HT, UK; william.jones-warner@lshtm.ac.uk (W.J.-W.); James.Ashall@lshtm.ac.uk (J.A.); Martin.Hibberd@lshtm.ac.uk (M.L.H.); Julius.Hafalla@lshtm.ac.uk (J.C.R.H.); 2 Department of Virology, Research Institute for Tropical Medicine, Manila 1781, Philippines; avakristysy@gmail.com (A.K.S.); maryannejoyreyes@gmail.com (M.A.J.R.); meannquinones@gmail.com (M.A.Q.); amado.tandocmd@gmail.com (A.O.T.); 3 Dengue Study Group, Research Institute for Tropical Medicine, Manila 1781, Philippines; lerosecap@yahoo.com.ph; 4 Department of Infectious Disease Epidemiology, Faculty of Epidemiology and Population Health, London School of Hygiene and Tropical Medicine, London WC1E 7HT, UK; Oliver.Brady@lshtm.ac.uk (O.J.B.); Adam.Kucharski@lshtm.ac.uk (A.J.K.); Sebastian.Funk@LSHTM.ac.uk (S.F.); 5 Centre for the Mathematical Modelling of Infectious Diseases, London School of Hygiene and Tropical Medicine, London WC1E 7HT, UK; 6 Department of Molecular Parasitology and Tropical Diseases, Graduate Institute of Medical Sciences, Taipei Medical University, Taipei 11031, Taiwan; jj45862001@gmail.com; 7 Department of Health, Philippine Epidemiology Bureau, Manila 1003, Philippines; flavelino.chd5@yahoo.com (F.L.A.); manemia_sucaldito@yahoo.com (N.L.S.); 8 Institute of Human Genetics, University of the Philippines, Manila 1000, Philippines; eccutiongcodelapaz@up.edu.ph (E.C.-d.l.P.); cdpadilla@up.edu.ph (C.D.P.); 9 Philippine Genome Centre, University of the Philippines, Manila 1101, Philippines

**Keywords:** Zika, dengue, serology, diagnostics, force of infection, Philippines

## Abstract

Zika virus (ZIKV) exposure across flavivirus-endemic countries, including the Philippines, remains largely unknown despite sporadic case reporting and environmental suitability for transmission. Using laboratory surveillance data from 2016, 997 serum samples were randomly selected from suspected dengue (DENV) case reports across the Philippines and assayed for serological markers of short-term (IgM) and long-term (IgG) ZIKV exposure. Using mixture models, we re-evaluated ZIKV IgM/G seroprevalence thresholds and used catalytic models to quantify the force of infection (attack rate, AR) from age-accumulated ZIKV exposure. While we observed extensive ZIKV/DENV IgG cross-reactivity, not all individuals with active DENV presented with elevated ZIKV IgG, and a proportion of dengue-negative cases (DENV IgG-) were ZIKV IgG-positive (14.3%, 9/63). We identified evidence of long-term, yet not short-term, ZIKV exposure across Philippine regions (ZIKV IgG+: 31.5%, 314/997) which was geographically uncorrelated with DENV exposure. In contrast to the DENV AR (12.7% (95%CI: 9.1–17.4%)), the ZIKV AR was lower (5.7% (95%CI: 3–11%)) across the country. Our results provide evidence of widespread ZIKV exposure across the Philippines and suggest the need for studies to identify ZIKV infection risk factors over time to better prepare for potential future outbreaks.

## 1. Introduction

Zika is a flavivirus predominantly transmitted by Aedes mosquitoes which typically causes asymptomatic, or occasionally mild self-limited symptomatic, infections in humans. Consequently, previous global Zika outbreaks during the 20th century were underreported, and the disease was of limited public health concern [1]. In 2016, Zika gained global prominence due to an outbreak in Brazil coinciding with an unprecedented rise in severe birth abnormalities [2]. Subsequent studies linked Zika virus infections with Guillain-Barré syndrome [3] and microcephaly in infants [4]. Today, heightened surveillance operations report evidence of autochthonous Zika transmission in approximately 87 countries [1]. However, population exposure rates and transmission patterns at subnational levels remain poorly characterized, at least partially because of the difficulties in distinguishing Zika from other flavivirus infections [5,6,7].

Similar to other flaviviruses, including dengue, Zika virus (ZIKV) infection in humans is characterized by an initial viremic, followed by an immunogenic phase. A few days post-infection, viremia increases rapidly in hosts, during which time viral RNA is detectable in the blood for a few days [8,9]. Shortly after this peak in viremia, hosts elicit IgM antibodies that likely persist for months post-infection [10]. Approximately a week after the peak in viremia, hosts mount a long-term IgG antibody response that offers protection from successive Zika infections and is thought to be detectable for decades [11]. In contrast, for flaviviral infections caused by dengue virus (DENV), the existence of four serologically distinct serotypes (DENV1-4) means that immunity only offers protection from subsequent homologous, not heterologous, serotypes enabling post-primary (secondary, tertiary, or quaternary) dengue infections [12]. During a secondary infection, previously elicited IgG no longer neutralizes, but instead cross-reacts and surges with the new serotype to trigger the antibody-dependent enhancement (ADE) of viral replication. Increased virus replication during a secondary infection is thought to result in more severe disease because host-elicited cytokine storms can trigger vascular leakage [12,13,14]. Interestingly, the extensive structural and antigenic homology between ZIKV and DENV has generated speculation as to whether cross-reactive IgG responses from a Zika infection can result in the enhancement of dengue [15]. Indeed, a recent cohort study conducted in Nicaragua revealed that infection by Zika enhances the future risk of severe disease in subsequent DENV-2 infections, comparable to a previous heterologous dengue serotype, suggesting possible ADE mechanisms [16]. A pattern also found in vivo when rhesus macaques, previously infected with ZIKV, experienced higher viremia and proinflammatory cytokines during a subsequent DENV-2 infection compared to those previously uninfected with ZIKV [17]. In contrast, a Brazilian study reported a decline in dengue infections following a Zika outbreak, eluding to cross-protection, not enhancement [18]. However, given that cross-protective ZIKV IgG antibodies wane over time [19], the remaining cross-reactive antibodies may facilitate adverse ADE mechanisms later in life.

With disease presentation largely asymptomatic and with a short window of viral detection, serological diagnosis is crucial for capturing Zika cases. However, cross-reactive antibody responses between ZIKV and DENV present a challenge for differential diagnosis. Numerous commercial serological diagnostic tests have been developed recently, including the Euroimmun (Lübeck, Germany) indirect IgM and IgG ELISAs (enzyme-linked immunosorbent assays), which state that the kits are highly specific for Zika [20]. Recent studies have utilized Euroimmun and have reported Zika specificity >90% [21,22], although the test subjects often included small groups of infected travelers who resided outside flavivirus-endemic countries. More recently, however, the accuracy of these commercial tests has been brought into question by studies in flavivirus-endemic regions, including Salvador (Brazil) [23], Rio de Janerio (Brazil) [24], and Carabobo (Venezuela) [25]. Studies have revealed that IgM ELISAs have adequate specificity, yet poor sensitivity for capturing active ZIKV infections. Conversely, studies have shown that ZIKV IgG ELISAs have favorable sensitivities, yet variable specificities in distinguishing Zika from dengue infections. Moreover, one group demonstrated that ZIKV IgG kits reasonably differentiated Zika infections from primary dengue infections, yet not secondary dengue infections [24], which may be a potential consequence of ZIKV IgG simply cross-reacting with pre-circulating IgG during a secondary infection elicited from either a prior dengue or Zika infection. This corresponds to findings described by the authors of [26], who evaluated a novel immunomagnetic assay for ZIKV and found that ZIKV IgG was elevated among secondary DENV infections, but not primary DENV infections. Further understanding into how ZIKV IgG responses change during the acute stage of a dengue infection, and of those reporting without active dengue infections, may help distinguish whether patients have experienced prior dengue or Zika infections.

In the Philippines, Zika transmission remains poorly understood. Prior to 2016, isolated reports of confirmed Zika infections among non-travelling individuals in two cities—Quezon City in 2010 [27] and Cebu City in 2012 [28]—eluded to autochthonous transmission rather than imported Zika. Then, in 2016, a total of 47 non-travelling, PCR-confirmed Zika cases were detected after enhanced surveillance operations incorporated fever, rash, arthralgia and conjunctivitis into their case definition [29]. Considering that Zika cases are often asymptomatic and symptomatic infections resemble other co-endemic febrile illnesses, relying on passive case reports likely underestimates the true burden of disease. In Thailand, a recent study revealed evidence of persistent Zika transmission throughout the whole country [6]. We therefore explored whether those reporting with suspected dengue across the Philippines, who are regularly sampled in accordance with existing laboratory surveillance, had evidence of recent or historical Zika exposure.

## 2. Methods

### 2.1. Flavivirus Surveillance in the Philippines

Zika and dengue are both notified at the point of care in health facilities, called disease reporting units (DRUs), across the Philippines in accordance with WHO and PIDSR (Philippine Integrated Disease Surveillance and Response) guidelines [30]. Suspected Zika case reports include those presenting with fever, conjunctivitis, skin rash and either of the following: headache, malaise, myalgia, malaise, joint pain, retro-orbital pain, travel to a Zika-reporting area, or a history of Guillain-Barré syndrome. Suspected cases also include infants/fetuses with neurological conditions with unknown etiologies, including reduced occipitofrontal circumference and/or intracranial calcifications. Serum, urine, and placental tissues collected from suspected cases undergo laboratory confirmation at the Research Institute for Tropical Medicine (RITM—Department of Health) and are assayed for anti-ZIKV PCR, IgM and IgG.

For dengue, suspected cases include those reporting with a sudden prolonged febrile illness accompanied by at least two additional symptoms: headache, body malaise, myalgia, arthralgia, nausea, vomiting, diarrhea, flushed skin and rash. All suspected dengue case reports are collated by the country’s Epidemiological Bureau. Additional laboratory surveillance, coordinated by the RITM, survey a representative of sample of suspected dengue cases and collect single serum samples at the point of care for further laboratory analysis. Five samples per week are randomly collected from patients who visit sentinel DRUs, which include major regional hospitals. Samples are also collected from those who visit non-sentinel DRUs following a surge of dengue case reporting in accordance with PIDSR criteria. Basic epidemiological data are collected from dengue patients: age, sex, date of symptom onset, date of reporting, health facility/home location (Region, Province, Barangay) and symptoms. Symptoms are categorized as no warning signs, warning signs (vomiting, fluid accumulation, mucosal bleeding, abdominal pain and liver enlargement) and severe dengue (severe plasma leakage, organ impairment and bleeding). Individuals excluded include those under 6 months or those who reported 5 days post-symptom onset.

### 2.2. Data Collection

For the purposes of this study, we selected a random subset of all dengue serum samples collected in 2016. In total, 1000 viable serum samples out of 3921 (25.5%) were selected and subjected for further Zika and dengue laboratory analysis. Additional covariates generated included disease day (date of symptom reporting—date of symptom onset), adverse clinical symptoms (severe dengue or warning signs) and urban barangay (>1500 persons per km^2^). Barangay population density refers to the 2015 barangay population over the barangay area (km^2^) (2015 Philippine census, Philippine Statistics Authority).

### 2.3. Laboratory Analysis

Patient serum samples were stored at −80 °C at the RITM prior to laboratory analysis. Using the semiquantitative Euroimmun™ indirect ELISA (Lübeck, Germany), we assayed samples for ZIKV IgM and IgG according to manufacturer’s instructions (Cat No: El 2668–9601 M and El 2668–9601 G). Output ratio values were subsequently termed ‘ZIKV IgM/G ELISA values’. ZIKV IgM and IgG ELISA values above 1.1 represented ZIKV IgM and IgG-seropositive samples, respectively. We also assayed samples for anti-DENV viremia, IgM and IgG. Using methods described by the authors of [31], a fourplex, real-time polymerase chain reaction (PCR) assay was used to detect serotype specific RNA to DENV1-4. An output critical threshold value below 36 was used to determine PCR-positive samples. For DENV IgM and IgG, Panbio™ capture indirect ELISA (Alere, Australia) kits were utilized in accordance with manufacturer guidelines (Cat. No: 01PE10 and 01PE20) to detect antibodies specific to any DENV serotype. Output index values were subsequently termed ‘DENV IgM/G ELISA values’. DENV IgM and IgG seroprevalence thresholds were previously generated by the authors of [32], corresponding to 0.99 and 0.22 ELISA units (Panbio index values), respectively.

### 2.4. Data Analysis

We determined individual DENV immune status according to the dengue laboratory and epidemiological data using methods previously described by the authors of [32]. Suspected dengue case reports were initially classified as active (PCR+ or IgM+) or non-active (PCR- and IgM-), as at least one of these markers should be present during an ongoing dengue infection. Active cases were further categorized as primary (IgG- on disease day 1–2 or IgG:IgM ratio < 0.45 on disease day 3–5) or post-primary (IgG+ on disease day 1–2 or IgG:IgM ratio > 0.45 on disease day 3–5). Post-primary cases included those with either secondary, tertiary or quaternary dengue infections with previous exposure to flaviviruses. Non-active dengue infections were further classified as historical (IgG+) or negative (IgG-) for dengue.

Mixture modelling methods were used to determine ZIKV IgM and IgG seroprevalence as described by the authors [32,33,34] using the ‘fmm’ command in STATA (v.16). Mixture models were fit to the ZIKV IgM/IgG ELISA value data by maximum likelihood with lognormal titer distributions and two components to characterize the seronegative and seropositive subpopulations. The existence of two components opposed to one subpopulation was justified according to Akaike information criterion (AIC), whereby lower AIC indicates superior model fit. Seroprevalence thresholds correspond to the lowest IgM/G ELISA values with >95% probability of being in the seropositive distribution.

Catalytic models were used to determine the DENV and ZIKV force of infection (and attack rates) across the Philippines among those without active ZIKV or DENV infections as antibody levels are heavily influenced by day of infection [11,32]. Models fitted with maximum likelihood were used to characterize anti-ZIKV/DENV IgG age-seroprevalence and generate seroconversion rates—estimates equivalent to the force of infection. For ZIKV, the seroconversion rate refers to the average annual rate at which the ZIKV-susceptible population (ZIKV IgG-) converts to ZIKV IgG+ status. For DENV, the seroconversion rate refers to the average annual rate at which the unexposed DENV IgG- population converts to DENV IgG+ status to any DENV serotype. Under the rationale that IgG wanes to low/undetectable levels with time, as shown by the authors of [35], we fitted both simple and reversible catalytic models in STATA using the ‘revcat’ command, which estimates the force of infection parameter using least squares. Consistent with individuals seroconverting to IgG+ following infection and remaining seropositive, Equation (1) estimates the probability of being seropositive by age (*a*) assuming constant force of infection (*λ*):

(1) $$P\left(a\right)=1-e^{-\lambda a}\;\;$$

In contrast, assuming individuals gradually lose IgG antibodies over time following infection, the reversible model (Equation (2)) fits an additional seroreversion parameter (*ρ*) to estimate the seroreversion rate: the average annual rate at which individuals serorevert back to IgG- status. AIC (Akaike information criterion) was used to determine whether simple or reversible catalytic models had the best model fit. To estimate the annual risk of ZIKV and DENV infection, FOI rate estimates were converted to attack rates (*AR*) according to Equation (3) [36].

(2) $$P\left(a\right)=\;\frac{\lambda }{\lambda +\rho }\left[1-e^{-\left(\lambda +\rho \right)a}\right]\;$$

(3) $$AR\;=\;1\;-\;e^{-\left(\lambda \right)}$$

Last, using univariate logistic regression modelling, we investigated whether those with post-primary DENV infections with/without ZIKV IgG exposure were as likely to present to clinics with adverse clinical and severe outcomes than primary DENV infections. We calculated the unadjusted odds ratios of presenting to DRUs with adverse clinical symptoms (warning signs of dengue or severe dengue) and severe dengue among those classified as post-primary dengue with/without Zika IgG exposure compared to primary dengue infections (ZIKV IgG-).

## 3. Results

### 3.1. Data Description

**Figure 1:**
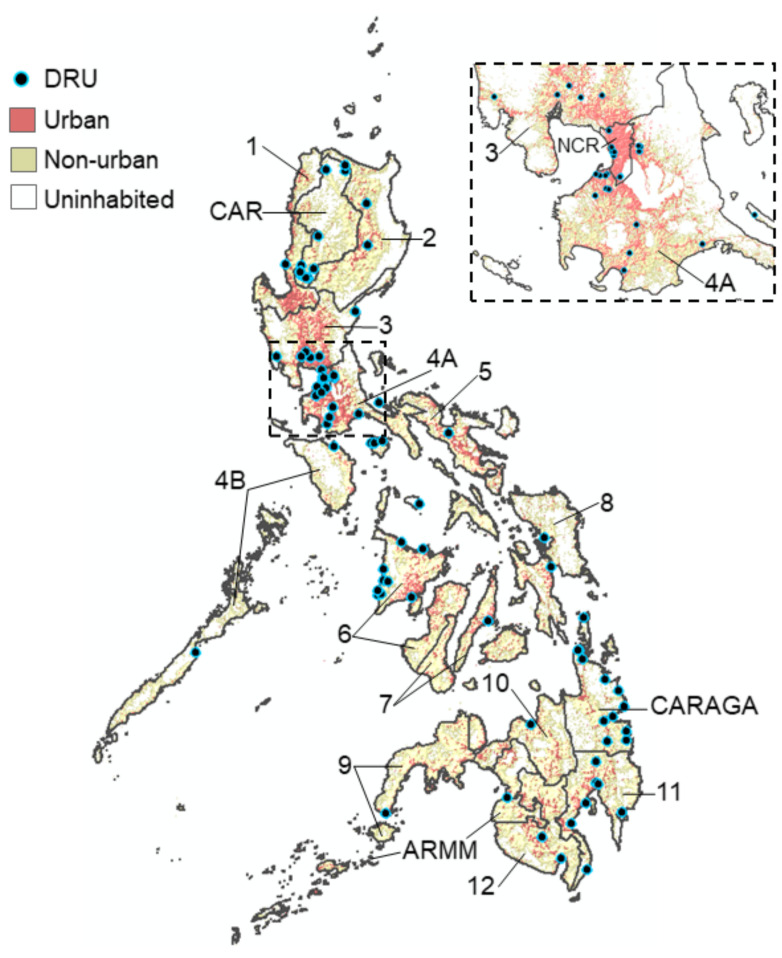
Map of the Philippines showing the location of the 102 DRUs where dengue patients were sampled from across all 17 regions during 2016. Urban zones: >1500 persons per km^2^. Non-urban zones: <1500 persons per km^2^.

We successfully assayed 997/1000 suspected dengue case reports who visited 102 DRUs situated in all 17 regions of the Philippines during 2016 (Figure 1) (Supplementary Materials Table S1). The demographic characteristics of the sampled population are shown in Supplementary Materials Table S2. Overall, most cases were aged between 6 and 15 years (43.3%, 432/997), reported between disease day four and five (51.4%, 512/997) and presented with warning signs of dengue (54.4% 542/997). Of those who reported with suspected dengue, we estimated that 80.1% (794/991) presented with an active DENV infection (PCR+ or IgM+). Among active dengue infections, we classified 23.8% (189/794) as primary and 76.2% (605/794) as post-primary infections.

### 3.2. ZIKV and DENV Cross-Reactive Antibody Responses

**Figure 2:**
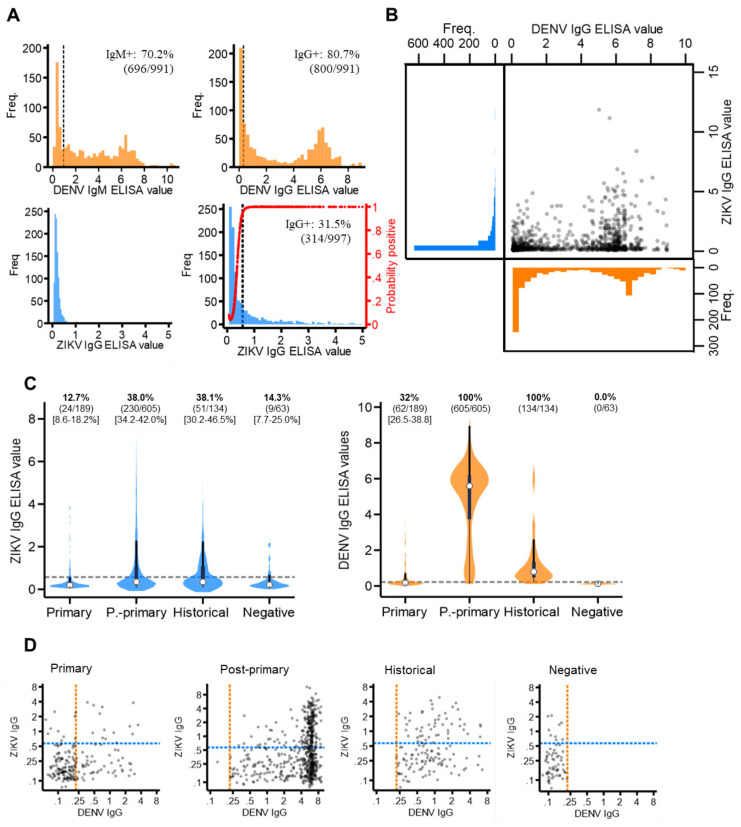
ZIKV and DENV antibody responses among the study population. (**A**) Distribution of ZIKV and DENV IgM and IgG antibody responses (ELISA values) among the study population. Red line: probability of being ZIKV IgG seropositive according to the mixture model. (**B**) Scatterplot of ZIKV versus DENV IgG ELISA values among the study population. (**C**) Violin plots of ZIKV and DENV IgG ELISA values among those classified as DENV primary, post-primary, historical and negative. White circles: median, thick black bar: IQR. Grey dash: IgG seroprevalence thresholds (ZIKV IgG: 0.57 ELISA values, DENV IgG: 0.22 ELISA values). (**D**) Scatterplots of ZIKV versus DENV IgG ELISA values among DENV primary, post-primary, historical and negative cases plotted on a log scale. Orange dash: DENV IgG seroprevalence threshold (0.22 ELISA values). Blue dash: ZIKV IgG seroprevalence threshold (0.57 ELISA values).

The majority of the sampled population reported with elevated dengue antibody responses, with 70.2% (696/991) DENV IgM+ and 80.7% (800/991) DENV IgG+ (Figure 2A). This infers a high degree of short- and long-term exposure to dengue among the sampled population.

For ZIKV IgM and IgG, seroprevalence thresholds were determined using mixture models. For ZIKV IgG, a two-component, as opposed to a one-component model, best fit the data (AIC difference: −284.2) (Supplementary Materials Figure S1). This generated a new ZIKV IgG seroprevalence threshold of 0.57 ELISA values, which resulted in 31.5% (314/997) of the study population having ZIKV IgG exposure (Figure 2A). For ZIKV IgM, a mixture model fitted with just one, instead of two, components best fit the data (AIC difference: +159.9), as most of the study population reported with very low ZIKV IgM levels. We were therefore unable to determine IgM seroprevalence. Thus, we concluded that, despite a proportion of the study reporting with long-term exposure, no evidence of recent Zika exposure was found in the study population.

Cross-reacting IgG responses between Zika and dengue are shown in Figure 2B. Among those with elevated (seropositive) DENV IgG, most reported with low levels of ZIKV IgG (63.1% (505/800) ZIKV IgG-). Contrastingly, among those with elevated (seropositive) ZIKV IgG, nearly all reported with elevated DENV IgG responses (93.6% (295/314) DENV IgG positive). This suggests the Panbio IgG ELISA kits detected IgG from either ZIKV or DENV, while Euroimmun IgG ELISA kits were more specific to ZIKV antibodies. Notably, after stratifying by DENV immune status, we found a significantly higher proportion of post-primary (38.0% (95%CI: 4.2–42.0%)) and historical (38.1% (95%CI: 30.2–46.5)) cases were classified as ZIKV IgG-positive compared to primary (12.7% (95%CI: 8.6–18.2%)) and negative (14.3% (95%CI: 7.7–25.0%)) dengue cases. Moreover, despite post-primary cases experiencing higher DENV IgG responses (median: 5.6 (IQR: 3.7–6.2)) than historical cases (median: 0.8 (IQR: 0.5–1.4)), the same proportion of these cases reported ZIKV IgG-seropositive (38%) (Figure 2C). Therefore, if ZIKV IgG was elevated solely due to elevated DENV IgG, then a higher proportion of post-primary than historical cases would be ZIKV IgG positive. However, this was not observed. Last, despite negative cases (clinically misdiagnosed dengue cases) being DENV IgG-negative, 14.3% (9/63) reported with distinctly elevated ZIKV IgG levels (ZIKV IgG-seropositive), which, notably, cannot be attributed to ZIKV/DENV cross-reactivity (Figure 2D).

### 3.3. ZIKV Immunoepidemiology in the Philippines

**Figure 3:**
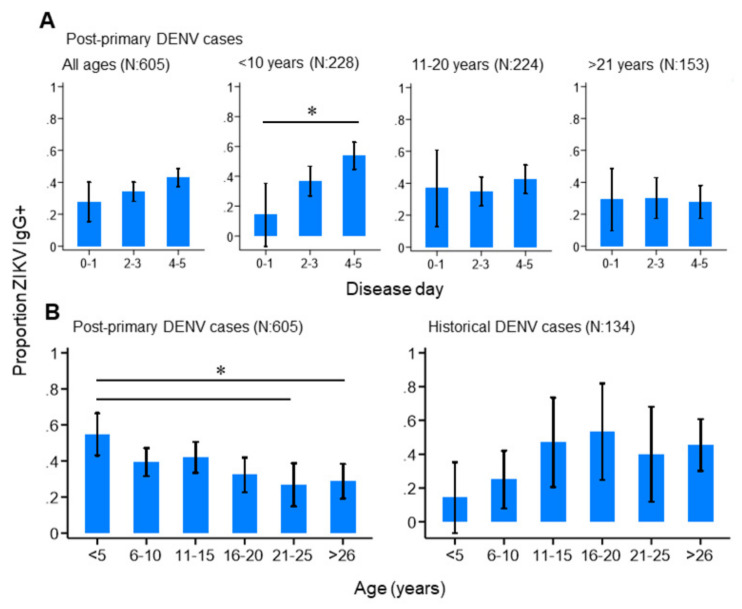
ZIKV IgG seroprevalence patterns among the study population. (**A**) ZIKV IgG seroprevalence by disease day among all, and age-stratified, post-primary dengue cases. (**B**) ZIKV IgG seroprevalence among post-primary and historical dengue cases. Vertical lines: 95%CI (confidence interval), (* non-overlapping 95%CI).

We next investigated how the progression of an active DENV infection influenced ZIKV IgG responses in the study population. Among those reporting with primary, historical or negative DENV infections, we observed no difference in the proportion ZIKV IgG positive by day of disease (Supplementary Materials Figure S2). This suggests that ZIKV IgG levels remained stable during the acute stage of primary DENV infections and in those reporting with historical or negative DENV infections. In contrast, ZIKV IgG positivity significantly increased by disease day among those reporting with post-primary DENV infections, particularly those under 10 years of age (Figure 3A). In total, 14.3% (95%CI: 0.00–35.3%) of post-primary infections under 10 years of age were ZIKV IgG-seropositive between disease days 0–1, which increased to 53.8% (95%CI: 44.7–62.9%) between disease days 4–5. Interestingly, this increasing trend was not observed among older post-primary infections (Figure 3A), but was observed among post-primary infections stratified by serotype, although these differences did not reach statistical significance (Supplementary Materials Figure S1). Together, this infers that ZIKV IgG surges during the acute phase of a post-primary DENV infection, particularly among those who are younger. We then explored how age impacted ZIKV IgG seroprevalence among the study population and found contrasting age-ZIKV IgG seropositivity trends between post-primary and historical DENV cases (Figure 3B). ZIKV IgG seroprevalence decreased with increasing age among post-primary DENV infections, whereby 54.8% (95%CI: 43.1–66.5%) of those under 5 years of age were ZIKV IgG-seropositive, which gradually decreased to 26.8% (95%CI 14.8–38.8%) among those aged between 21–25 years. ZIKV IgG seroprevalence appeared to increase with age among historical cases, although this was not statistically significant (Figure 3B). Taken together, these results show that younger, as opposed to older, age post-primary DENV infections had high levels of ZIKV IgG which surged rapidly during the acute stage of disease.

**Figure 4:**
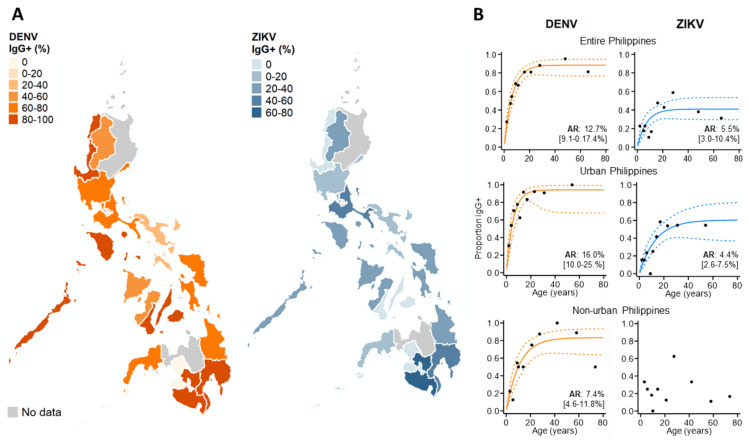
Immunoepidemiology of ZIKV and DENV across the Philippines during 2016. (**A**) Regional ZIKV/DENV IgG seroprevalence among those reporting without active DENV infections. (**B**) ZIKV/DENV age seroprevalence across the Philippines and stratified by urban/non-urban areas. ARs (attack rates) were calculated from the seroconversion rate (SCR) estimated among those without current DENV infections (historical and negative DENV cases) using reverse catalytic models. Black dots: observed age IgG seroprevalence. Curve: predicted age IgG seroprevalence. Dash: 95%CI.

We then explored the spatial patterns in ZIKV and DENV IgG exposure across the Philippines in 2016 among those without active dengue infections, as ZIKV IgG is impacted by changing levels of DENV IgG during an active DENV infection (Figure 4A). We found that ZIKV historical exposure was widespread across the Philippines, with ZIKV IgG+ individuals identified in 15/17 Philippine regions. Moreover, we observed no statistical correlation between regional ZIKV and DENV IgG exposure, inferring that elevated ZIKV IgG is not attributed to higher DENV IgG (ρ: 0.26, *p*-value: 0.184) (Supplementary Materials Figure S3). We also found further evidence of widespread ZIKV exposure, identifying seven regions across the Philippines where DENV-negative (DENV IgG-) cases reported as ZIKV IgG-seropositive (Supplementary Materials Table S3). However, numbers were small (a total of 63 negative dengue cases in 11/17 regions across the Philippines).

To investigate the DENV and ZIKV annual attack rate, we used catalytic models to characterize age seroprevalence among those reporting without active DENV infections (DENV PCR- & IgM-). According to AIC, reversible, as opposed to simple catalytic models, had superior model fits. For dengue, increasing DENV IgG seroprevalence with age generated an AR estimate which suggests that 12.7% (95% CI: 9.1–17.4%) of the study population became exposed to DENV annually. DENV AR estimates were slightly higher in urban settings (AR: 16.0% (95%CI: 10.0–25.0%)) and lower in non-urban settings (7.4% (95%CI: 4.6–11.8%)). For Zika, the overall AR was lower than for dengue, with an estimated 5.5% (95%CI: 3.0–10.4%) of the study population becoming exposed annually. After stratifying by population density, the Zika FOI remained similar in urban centers across the Philippines. In contrast, we observed no increasing age ZIKV IgG seroprevalence in non-urban areas and were unable to estimate the FOI (Figure 4B).

Last, we explored whether post-primary dengue cases with/without prior ZIKV IgG exposure had a similar risk of adverse clinical/severe symptoms compared to primary DENV infections (Table 1). Adverse clinical symptoms included reporting with either warning signs of dengue or severe dengue disease. Among the 81.6% (814/997) of the study population with symptom data, 73.3% of primary DENV infections, 80.7% of post-primary DENV infections without ZIKV IgG exposure and 87.4% of post-primary DENV infections with ZIKV IgG exposure presented with adverse clinical symptoms. Moreover, compared to primary DENV infections, post-primary infections with prior Zika exposure were statistically more likely to experience adverse clinical outcomes (OR: 2.52 (95%CI: 1.42–4.49), *p*-value: 0.002). However, no such association was identified upon stratifying the outcome by just severe disease. Post-primary DENV infections with prior exposure to Zika did not have a significantly higher risk of presenting with severe disease compared to primary infections (OR: 1.31 (95% CI: 0.66–2.60), *p*-value: 0.438). This pattern is likely attributed to severe dengue being a rare disease outcome. It should also be noted that, among the study population with active disease, those with elevated DENV IgG, on average, had higher ZIKV IgG. Moreover, those with active disease and elevated DENV IgG were more likely to present with adverse clinical symptoms (Supplementary Materials Figure S4).

## 4. Discussion

Our results show that, during 2016, suspected dengue cases from the Philippines had evidence of long-term, yet not short-term, serological exposure to Zika. This suggests that widespread ZIKV epidemiological investigations are warranted to determine the future risk of disease outbreaks across the country. We confirmed substantial IgG cross-reactivity between ZIKV and DENV, particularly among those reporting with post-primary DENV infections, where ZIKV IgG responses increased with disease progression. Among those reporting with non-active DENV infections (clinically misdiagnosed dengue cases), however, ZIKV IgG levels remained constant by reported day of disease, and some of those without any evidence of DENV IgG still had elevated ZIKV IgG. Last, we showed that the Zika FOI was lower than the dengue FOI across the Philippines, and that Zika exposure accumulated with age across urban settings, suggesting persistent transmission.

During the early stages of a secondary dengue infection, hosts experience a storm of specific and non-specific DENV IgG originally elicited from a previous, heterologous, serotype infection [12,13,14]. In our study, we revealed significant ZIKV/DENV assay cross-reactivity among those presenting with post-primary dengue infections. Our findings are consistent with those previously reported in Brazil, which found that Euroimmun kits are capable of distinguishing Zika infections from primary, yet not secondary, dengue infections [24]. We suggest two factors that account for this finding. First, Zika kits may simply detect pre-circulating, non-specific DENV IgG during a post-primary dengue infection that originated from a prior heterologous DENV serotype due to cross-reactivity. Alternatively, kits may detect IgG in post-primary dengue infections that was elicited from a prior Zika, not dengue, infection which cross-reacted with the subsequent dengue infection to mimic secondary-like disease. The latter is consistent our findings that ZIKV IgG seroprevalence increased with disease progression during post-primary infections, suggesting ZIKV IgG-induced ADE mechanisms. Moreover, we found that only a subset of those with post-primary DENV infections had elevated ZIKV IgG. Furthermore, we found that post-primary dengue cases with ZIKV IgG exposure, like post-primary DENV infections without ZIKV exposure, were at higher risk of presenting with adverse clinical symptoms than primary infections, a trend similarly reported by the authors in [16], and possibly a consequence of prior ZIKV exposure, priming individuals for a worse secondary-like dengue infection. However, considering that those with elevated DENV IgG were more likely to have higher ZIKV IgG and that elevated DENV IgG is associated with severe disease outcomes, this association may be confounded by cross-reactivity. Interestingly, we showed that younger reporting post-primary cases were more likely to be ZIKV IgG-positive compared to older age post-primary DENV infections. We speculate that younger post-primary DENV infections include more secondary DENV infections while older age post-primary DENV infections include more post-secondary infections (tertiary/quaternary dengue), as older individuals are more likely to have experienced more than two DENV infections in their lifetime [37]. Moreover, secondary DENV infections experience larger surges in IgG levels compared to post-secondary infections due to ADE mechanisms [13]. Therefore, younger post-primary DENV infections, which likely included more secondary, as opposed to post-secondary, infections, were more likely to be ZIKV IgG-positive due to ZIKV IgG ELISAs detecting non-specific surging IgG responses compared to older post-primary infections with subdued IgG responses. Consequently, we were unable to conclusively determine whether ZIKV IgG exposure among post-primary infections was due to true prior exposure or simply cross-reactivity. Novel IgG assays that truly distinguish cross-reactive antibodies from true exposure are needed to overcome these limitations [26].

Following a dengue infection, heightened IgG levels wane, leaving hosts with DENV-specific IgG that persists for decades at lower levels [12]. Consequently, we speculated that historical and negative dengue cases reporting with elevated ZIKV IgG truly experienced a prior a ZIKV infection. We attribute this to two factors. First, only a subset of historical and negative cases presented with elevated ZIKV IgG. If the Euroimmun kits were also detecting DENV-specific IgG, then those with elevated DENV IgG would have elevated ZIKV IgG. Second, some of those reporting with negative dengue infections, without evidence of any DENV IgG, were ZIKV IgG-positive, which cannot be attributed to cross-reactivity. Among those reporting with non-active DENV infections, we revealed evidence of widespread ZIKV exposure across the Philippines similar to previous findings in Thailand [6]. This suggests that focal Zika surveillance practices in the Philippines would likely miss Zika infections and justifies further investigations into Zika transmission dynamics across the country.

Across the Philippines, the ZIKV FOI was lower than for DENV FOI, as expected, given the huge difference in reported cases. However, considering that DENV Panbio IgG ELISAs also detect ZIKV IgG, similarly shown by the authors of [23], and our study population included reported patients, our DENV FOI estimate is likely slightly overestimated. However, after stratifying by population density, we found that DENV transmission intensity was higher in urban compared to non-urban settings, as demonstrated previously in Bangladesh [38]. Moreover, accumulating DENV exposure with age in urban and non-urban areas eludes to well-established, historical dengue transmission in both these settings [7]. For ZIKV, however, we found that FOI in urban areas was very similar to the overall country. However, in non-urban areas, there was no evidence of increasing ZIKV exposure with age. This is consistent with the rationale that historical, or potentially ongoing ZIKV transmission, is more common in urban settings. We suggest two factors that may account for this finding. First, ZIKV transmission in the Philippines is more recent than DENV and is still only dominant in urban areas where transmission originated [39]. Second, widespread DENV exposure across the Philippines offers the population protection from ZIKV, as suggested by the authors of [7], and hampers the spread of ZIKV into more rural areas. The observation that ZIKV seroprevalence is higher in younger individuals in non-urban settings also suggests more recent ZIKV outbreaks. However, this requires further epidemiological validation.

No evidence of recent ZIKV exposure among the study population was observed in this study, which we believe is attributed to several factors. Earlier studies have shown ZIKV outbreaks to be periodic in nature [7,16,40]. Therefore, we may have collected samples during a non-outbreak period. However, this contradicts with DOH reports of laboratory-confirmed cases of Zika across the country during 2016. Second, Zika is thought to be a largely asymptomatic infection, so most of those infected would be unlikely to seek care. Third, as only 997 dengue case reports were sampled across the country and Euroimmun IgM ELISAs have previously shown to suffer low sensitivity [22], we may have missed recent ZIKV infections. Therefore, future surveillance and epidemiological programs should consider the type of samples that should be collected from individuals and what laboratory procedures could be used to maximize the chances of identifying those with recent ZIKV infections. A recent study showed that ZIKV RNA and IgM compartmentally persist in hosts and that novel diagnostic methods might extend the window of detection [9]. Despite not capturing recent Zika infections, our study still revealed evidence of long-term exposure to Zika. Therefore, we believe that further epidemiological studies into ZIKV transmission across the Philippines are warranted. Population-based seroprevalence studies would provide better understanding into the spatiotemporal nature of ZIKV transmission across the country and identify regions with or without the disease. Moreover, despite not capturing recently reported ZIKV infections in this study, routinely assaying suspected DENV cases for ZIKV, particularly those without active DENV infections, may still assist in identifying future outbreaks.

## 5. Conclusions

In this study, we provided the first evidence of widespread ZIKV exposure across the Philippines and suggest ZIKV transmission has potentially been ongoing in urban areas for many years. Despite detecting cross-reactivity between DENV and ZIKV IgG responses, our analysis provides evidence of ZIKV transmission by considering dengue serological findings. Our results highlight the need for continued investigations into ZIKV transmission across the Philippines and justify combining ZIKV surveillance with other flaviviruses. Together, this could better describe ZIKV exposure over time and help curb possible future outbreaks of severe outcomes associated with ZIKV.

## Figures and Tables

**Table 1:** Clinical manifestations associated with primary DENV infections (ZIKV IgG-), post-primary DENV infections (ZIKV IgG-) and post-primary DENV infections (ZIKV IgG+). Adverse clinical symptoms: dengue warning signs or severe symptoms. OR: odds ratios.

Reported DENV/ZIKV	N	Adverse Clinical Symptoms				Severe Symptoms			
Immune Status		%	OR	[95% CI]	*p*-Value	%	OR	[95% CI]	*p*-Value
Primary DENV	131	73.3	1 (ref)			9.2	1 (ref)		
(ZIKV IgG-)									
Post-primary DENV	306	80.7	1.53	[0.94–2.47]	0.084	6.9	0.69	[0.33–1.34]	0.254
(ZIKV IgG-)									
Post-primary DENV	190	87.4	2.52	[1.42–4.49]	0.002	12.6	1.31	[0.66–2.60]	0.438
(ZIKV IgG+)									

## Data Availability

Data are available from the corresponding author upon reasonable request.

## Supplementary Materials

The following are available online at https://www.mdpi.com/article/10.3390/v13081441/s1, Table S1: Regional administrative boundaries of the Philippines, Table S2: Demographic characteristics of the study population, Figure S1: Methods used to determine ZIKV IgM and IgG seroprevalence, Figure S2: ZIKV IgG seroprevalence, Figure S3: Scatter plot of regional DENV versus ZIKV IgG seroprevalence among those with non-active DENV infections, Table S3: The percentage of reporting negative DENV cases (DENV PCR-, IgM- and IgG-) across the Philippine regions who were ZIKV IgG positive, Figure S4: Cross-reactive ZIKV/DENV IgG responses.
