# Supplementary material for: Serological Evidence of Widespread Zika Transmission across the Philippines
Source: Viruses. 2021 Jul 23;13(8):1441. doi: 10.3390/v13081441 (PMC8402696; doi:10.3390/v13081441)
Supplement: Supplementary file 1 [file viruses-13-01441-s001.zip › viruses-1298009-supplementary.pdf]

Supplementary materials

Title: Serological evidence of widespread Zika transmission across the Philippines

Table S1: Regional administrative boundaries of the Philippines.

Island group	Region	
	Code	Name
Luzon	1	Ilocos Region
	CAR	Cordillera Administrative Region
	2	Cagayan Valley
	3	Central Luzon
	4A	Calabarzon
	4B	Mimaropa
	5	Bicol Region
	NCR	National Captial Region
Visayas	6	Western Visayas
	7	Central Visayas
	8	Eastern Visayas
Mindanao	9	Zamboanga Peninsula
	10	Northern Mindanao
	11	Davao Region
	12	Soccsksargen
	CARAGA	Caraga Region
	ARMM	Autonomous Region in Muslim Mindanao

Table S2: Demographic characterisitcs of the study population.

Study demographics		
	%	n
**Age**		
<5	16.5	165
6-15	43.3	432
16-30	27.8	277
>30	12.3	123
**Sex**		
Female	48.6	485
Male	51.4	512
**Disease day**		
0-1	8.8	88
2-3	39.8	397
4-5	51.4	512
**DENV symptoms**		
No warning signs	17.5	174
Warning signs	54.4	542
Severe dengue	9.8	98
Non-disclosed	18.4	183
**Island group**		
Luzon	48.5	484
Visayas	17.5	174
Mindanao	34.0	339
**DENV immune status**		
Primary	19.1	189
Post-primary	61.0	605
Historical	13.5	134
Negative	6.4	63
**Total**	100	997

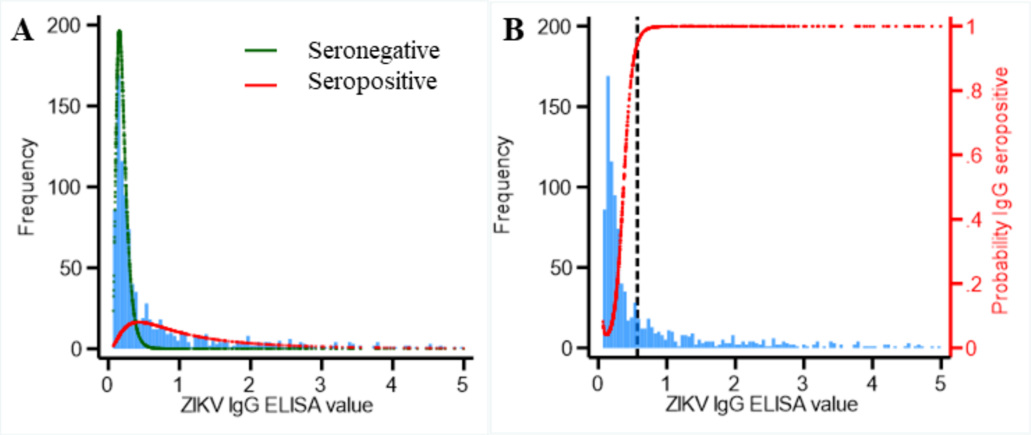

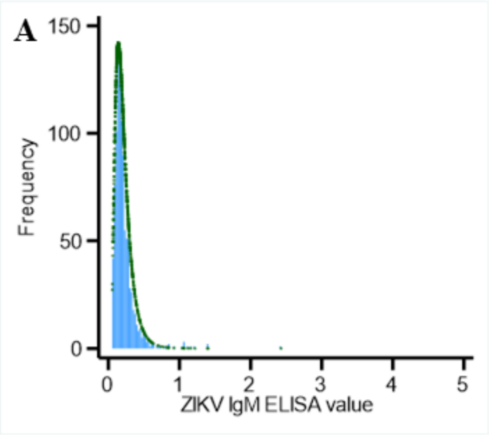

C

IgG model Component	AIC	AIC difference
1	920.5	
2	636.3	-284.2

IgM model Component	AIC	AIC difference
1	876.2	
2	1036.1	+159.9

Figure S1: Methods used to determine ZIKV IgM and IgG seroprevalence. A: Histogram plots of the study populations ZIKV IgM and IgG distributions fitted with two mixture model subpopulations: green: seronegative, red: seropositive. B: Histogram of ZIKV IgG overlaid by the probability of being seropositive according the mixture model. Vertical dash (revised cut off): >95% probability of IgG seropositive to ZIKV. C: Model fit comparison of ZIKV IgM and IgG distributions according to AIC.

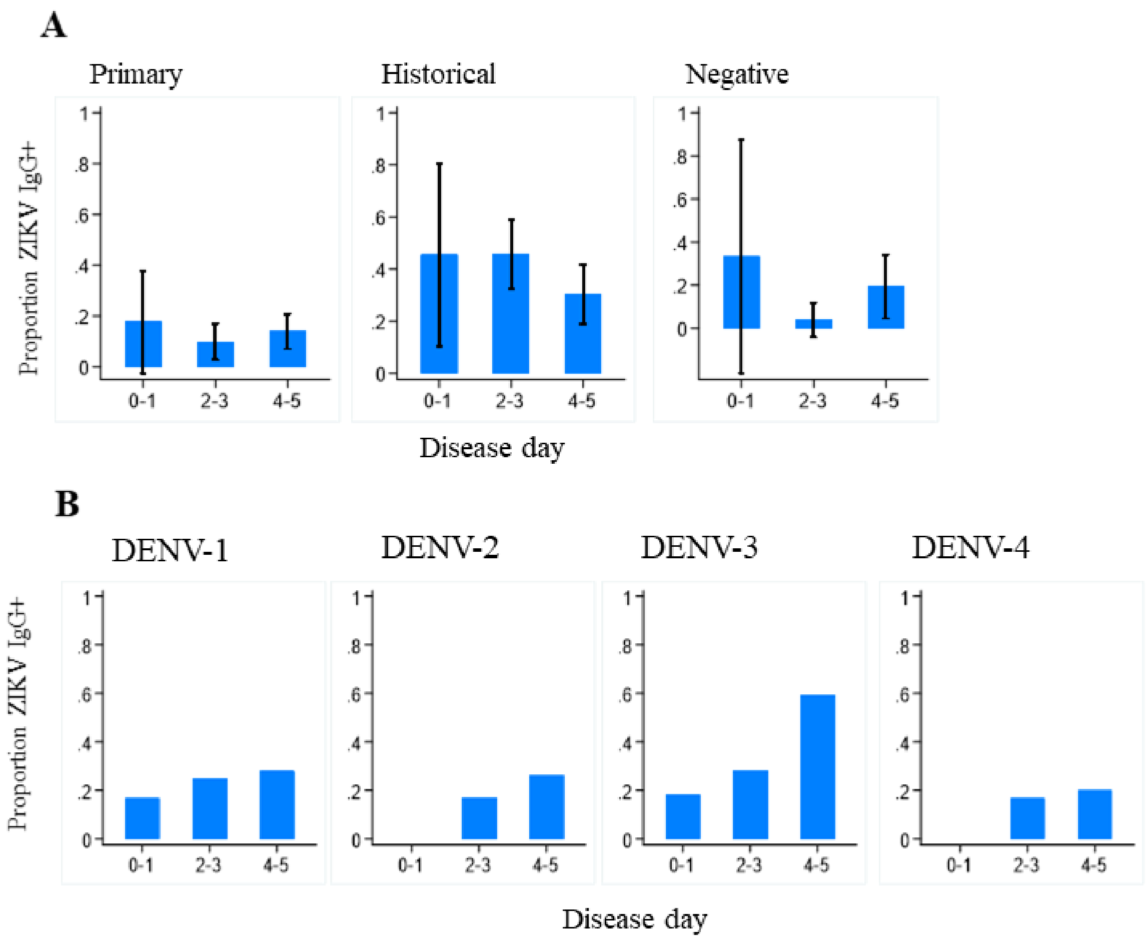

Figure S2: A: ZIKV IgG seroprevalence by day of disease among those reporting with primary, historical and negative dengue infections. B: ZIKV IgG seroprevalence by day of disease among those reporting with DENV1-4 infections.

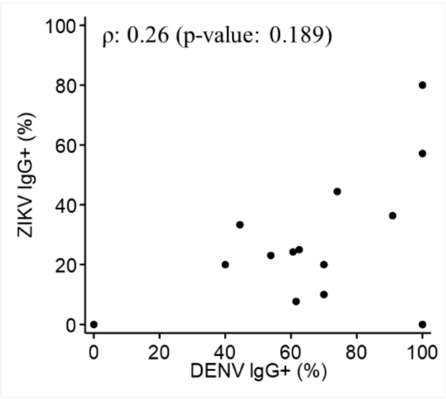

Figure S3: Scatter plot of regional DENV versus ZIKV IgG seroprevalence among those with non-active DENV infections. Rho: Pearson's R coefficient.

Table S3: The percentage of reporting negative DENV cases (DENV PCR-, IgM- and IgG-) across the Philippine regions who were ZIKV IgG positive.

Region	N	ZIKV IgG seropositive	
		%	n
3	5	0.0	0
4A	14	7.1	1
4B	1	100.0	1
5	3	33.3	1
6	5	0.0	0
8	3	66.7	2
9	3	33.3	1
ARMM	1	0.0	0
CAR	12	8.3	1
CARAGA	13	15.4	2
NCR	3	0.0	0
Total	63	14.3	9

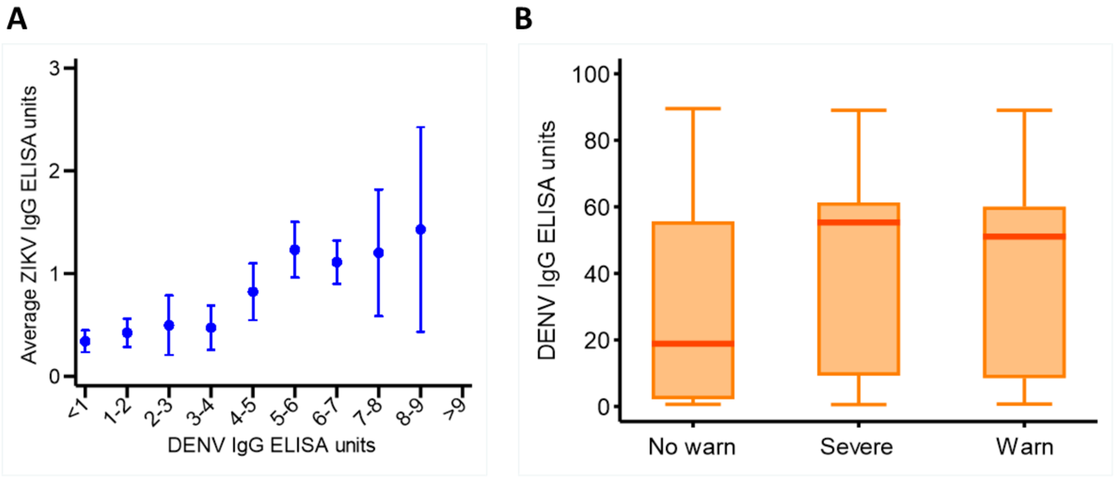

Figure S4: Cross-reactive ZIKV/DENV IgG responses. A: Average ZIKV IgG ELISA value over stratified DENV IgG ELISA values. Vertical bars: 95%CIs. B: DENV IgG responses among active dengue infections presenting with warning signs (warn), severe dengue (severe) and no warning signs (no warn).
